# Editorial for the Special Issue on Structural Analyses and Designs for Flexible/Stretchable Electronics

**DOI:** 10.3390/mi14061211

**Published:** 2023-06-08

**Authors:** Rui Li

**Affiliations:** State Key Laboratory of Structural Analysis, Optimization and CAE Software for Industrial Equipment, Department of Engineering Mechanics, International Research Center for Computational Mechanics, Dalian University of Technology, Dalian 116024, China; ruili@dlut.edu.cn

Flexible/stretchable electronics constitute a class of prospective technology incorporating stretchable/bendable/twistable components such that unprecedented properties superior to those of conventional rigid/brittle electronics are realized over. The modeling, design, and fabrication of flexible/stretchable electronics have attracted tremendous scholarly attention due to their wide applications to many emerging devices, including flexible displays, conformable sensors, epidermal electronics, implantable transient electronics, etc. To facilitate the more rapid development and broader applications of flexible/stretchable electronics, this Special Issue sought contributions on structural analyses and designs of flexible/stretchable electronics with different component-level or device-level applications, with topics including theoretical, numerical, and experimental studies.

In this Special Issue, there are 16 papers, of which 14 are research articles and 2 are review articles. Twelve articles are by authors from China and one paper each was contributed by authors from Germany, Italy, Poland, and Russia. A comprehensive range of aspects related to the structural analyses, designs, and fabrication of flexible/stretchable devices and smart materials are covered, including mechanical analyses of flexible/stretchable devices [1,2,3,4], thermal analyses of flexible heaters [5,6,7,8,9], mechanical–conductive analyses of island-type structures in flexible/stretchable devices [10], thermo-mechanical analyses and designs of morphing aircraft structures [11], adhesive bonding technology for flexible substrates [12], electrohydrodynamic jet printing technology for flexible substrates [13], process parameters’ influence on the resistance of 3D-printed electrically conductive structures [14], and recent advances in flexible radio frequency microelectromechanical systems (RE MEMS) [15] and triboelectric nanogenerators (TENGs) [16].

The analyses of flexible/stretchable structures used in different physical fields were implemented to investigate their characteristics, manipulating the mechanical, thermal, and conductive properties. Specifically, Yu et al. [1] developed a highly accurate reconstruction method for the large deflection of smart deformable structures, in which the fixed-flexible nozzle plate was taken as the subject. Groth et al. [2] presented a radial basis functions meshless approach to evaluate strain due to large displacements in flexible printed circuit boards, showing satisfactory comparability with the finite element method (FEM). Ji et al. [3] established a mathematical model incorporating skin compression, transduction, modulation, and perception for skin pain sensation under locally distributed mechanical compression for electronic skin applications, showing good agreement with the FEM. Li et al. [4] employed the shooting method to analyze the finite-deformation-theory-based mechanical behaviors of origami-inspired horseshoe-shaped solar arrays, followed by their counterparts with the FEM for comparison. He et al. [5,6] conducted a study on analytic models for the orthotropic transient heat conduction of stretchable rectangular and network heat sources to investigate the heat flux manipulation mechanism and the temperature distribution homogenization effect in epidermal electronic systems. Zhao et al. [7] developed a Fourier-cosine-transform-based analytic orthotropic heat conduction model with a complex serpentine heat source, which was validated using the FEM, and investigated the effects of geometric spacing and orthotropic thermal conductivity on the thermal properties. Li et al. [8] conducted systematic thermal and mechanical analyses of a kind of flexible microheater with two different wire structures and provided structural design guidelines to obtain high-efficiency flexible microheaters for different applications. Xu et al. [9] proposed an analytic model of transient heat conduction for bi-layered flexible electronic heaters via symplectic superposition and Laplace transformation and further analyzed the thermal conductivity ratios’ effect on the conduction process. Slepchenkov et al. [10] studied the mechanical and conductive behaviors of graphene–carbon nanotube hybrid films with an island topology under axial deformation using the self-consistent charge density functional tight-binding method.

In addition to structural analyses of flexible/stretchable electronics, several design theories and fabrication technologies are also reported in this Special Issue. Zhang et al. [11] implemented the UMAT in ABAQUS to realize the novel design of a morphing skin with a shape memory alloy based on an equivalent thermal stress approach and investigated the corresponding experiments, with consistent counterpart results. Saleh et al. [12] studied the isotropic conductive adhesive and the solder and lifetime characterization of an assembly of surface-mounted devices on flexible substrates and summarized the better adhesive surfaces for future structural designs. Wang et al. [13] reported on the simulation and printing of microdroplets using a straight electrode-based electrohydrodynamic jet for the flexible substrate, proving the feasibility and high merit of this fabrication technology. Dembek et al. [14] investigated the influences of printing parameters including the print temperature, layer height, nozzle diameter, and extrusion rate on the resistance of 3D-printed conductive composite structures and provided essential guidance on printing parameters to reduce the resistance of printed paths for structural electronics.

Furthermore, the review articles in this Special Issue report on the wider advances in recent innovative research in this field. Shi and Shen [15] provided an overview of recent achievements in the structural design, fabrication process, and performance optimization of flexible RF MEMS and recommended several future research directions with breakthrough potential. Zhao et al. [16] reviewed the TENG-based self-powered systems, together with their high-performance energy conversion, high-sensitivity detection of mechanical stimulation, and reliable output strategies, in harsh environments and addressed the challenges and barriers to the practical application of TENGs.

In conclusion, this Special Issue on “Structural Analyses and Designs for Flexible/Stretchable Electronics” will provide readers with a comprehensive understanding of the latest advancements in the field, as well as an introduction to the cutting-edge techniques that have recently emerged.
